# Prescribing of psychotropic medications to the elderly population of a Canadian province: a retrospective study using administrative databases

**DOI:** 10.7717/peerj.168

**Published:** 2013-09-17

**Authors:** Silvia Alessi-Severini, Matthew Dahl, Jennifer Schultz, Colleen Metge, Colette Raymond

**Affiliations:** 1 Faculty of Pharmacy, University of Manitoba, Winnipeg, Manitoba, Canada; 2 Manitoba Centre for Health Policy, University of Manitoba, Manitoba, Canada

**Keywords:** Antipsychotic, Benzodiazepines, Elderly, Prescribing, Psychotropic

## Abstract

**Background.** Psychotropic medications, in particular second-generation antipsychotics (SGAs) and benzodiazepines, have been associated with harm in elderly populations. Health agencies around the world have issued warnings about the risks of prescribing such medications to frail individuals affected by dementia and current guidelines recommend their use only in cases where the benefits clearly outweigh the risks. This study documents the use of psychotropic medications in the entire elderly population of a Canadian province in the context of current clinical guidelines for the treatment of behavioural disturbances.

**Methods.** Prevalent and incident utilization of antipsychotics, benzodiazepines and related medications (zopiclone and zaleplon) were determined in the population of Manitobans over age 65 in the time period 1997/98 to 2008/09 fiscal years. Comparisons between patients living in the community and those living in personal care (nursing) homes (PCH) were conducted. Influence of sociodemographic characteristics on prescribing was assessed by generalized estimating equations. Non-optimal use was defined as the prescribing of high dose of antipsychotic medications and the use of combination therapy of a benzodiazepine (or zopiclone/zaleplon) with an antipsychotic. A decrease in intensity of use over time and lower proportions of patients treated with antipsychotics at high dose or in combination with benzodiazepines (or zopiclone/zaleplon) was considered a trend toward better prescribing. Multiple regression analysis determined predictors of non-optimal use in the elderly population.

**Results.** A 20-fold greater prevalent utilization of SGAs was observed in PCH-dwelling elderly persons compared to those living in the community. In 2008/09, 27% of PCH-dwelling individuals received a prescription for an SGA. Patient characteristics, such as younger age, male gender, diagnoses of dementia (or use of an acetylcholinesterase inhibitor) or psychosis in the year prior the prescription, were predictors of non-optimal prescribing (e.g., high dose antipsychotics). During the period 2002/3 and 2007/8, amongst new users of SGAs, 10.2% received high doses. Those receiving high dose antipsychotics did not show high levels of polypharmacy.

**Conclusions.** Despite encouraging trends, the use of psychotropic medications remains high in elderly individuals, especially in residents of nursing homes. Clinicians caring for such patients need to carefully assess risks and benefits.

## Introduction

Antipsychotic medications have been prescribed to elderly persons mainly for the treatment of behavioural disturbances of dementia (Chan, Lam & Chen, 2011; Leslie & Rosenheck, 2012; Lee et al., 2004; Tariot, 2004). The safety and effectiveness of both first-generation (FGAs; e.g., haloperidol and phenothiazines) and second-generation antipsychotic agents (SGAs; e.g., risperidone, olanzapine, quetiapine) have been questioned and severe adverse events, including death, have been reported in elderly patients treated with antipsychotics in both RCTs and observational studies (Ray et al., 2001; Percudani et al., 2005; Wang, Schneeweiss & Avorn, 2005; Schneeweiss et al., 2007; Trifiro et al., 2007; Mehta et al., 2010; Sacchetti, Turrina & Valsecchi, 2010; Mittal et al., 2011; Vasilyeva et al., 2013). Because of the strong evidence of harm caused by these agents to elderly patients affected by dementia, Health Canada and other health agencies worldwide issued several warnings between 2002 and 2005 (Health Canada, 2002; Health Canada, 2004; Health Canada, 2005; The Food and Drug Administration, 2008; European Medicines Agency (EMEA), 2009). Benzodiazepines (BZDs) and other psychotropic medications have also been associated with harm (e.g., increased risk of falls) in older adults (Eigenbrodt et al., 2000; Ayer et al., 2007; Barlett, Abrahamowicz & Grad, 2009; Hill & Wee, 2012), and evidence-based practice guidelines advise clinicians to consider all factors before prescribing potent medications to frail individuals (APA Practice Guidelines, 2007; Peisah, Chan & McKay, 2011; Jeste, Blazer & Casey, 2008; Gauthier, Patterson & Chertkow, 2012). The objectives of this study were:

1. to evaluate the use of psychotropic medications (i.e., antipsychotics and benzodiazepines) in the elderly population of a Canadian province in light of health agency warnings and optimal prescribing guidelines;
2. to determine predictors of non-optimal use of psychotropic medication in elderly persons.

## Methods

This retrospective population-based study received ethics approval from the Research Ethics Board of the University of Manitoba. The study was conducted in full compliance with the Personal Health Information Act of Manitoba (Personal Health Information Act of Manitoba, 2013) and approved by the provincial Health Information Privacy Committee.

Data for the study were obtained from the administrative health care databases of the Manitoba Population Health Research Data Repository, housed at the Manitoba Centre for Health Policy. The databases include de-identified information on the entire population of the province and the use of a consistent set of quasi-identifiers permits the building of health histories of non-identifiable individuals across files and time. Nearly all contacts with the universal provincial health care system, including physicians, hospitals, personal care (nursing) home (PCH) residence, and pharmaceutical dispensations are recorded. All registered individuals possess a 9-digit personal health identification number (PHIN), which is encrypted to protect privacy. The following databases were accessed: (1) population registry, (2) hospital abstracts, (3) medical services, (4) Drug Product Information Network (DPIN) prescription records, (5) PCH records, (6) vital statistics and (7) prescriber characteristics.

Records of physician reimbursement for medical care (ambulatory care visits and hospitalizations) are submitted under a fee-for-service arrangement and contain information on patient diagnosis based on the International Classification of Diseases, Clinical Modification (ICD-9-CM and ICD-10-CM codes) (International Classification of Diseases, 2013). Records of dispensed prescriptions (DPIN) available through retail pharmacies contain data on the date of dispensing, drug name, strength, dosage form, and quantity, and the 8-digit drug identification number (DIN). Diagnoses can be retrieved from the hospital abstracts and medical services databases and linked with the population registry and vital statistics for demographic characteristics of patients and dates of death. Prescriber characteristics focus on the prescriber who wrote the incident prescription.

Region of residence (urban vs. rural) was determined by the postal codes registered with Manitoba Health. The distribution of urban (major cities of the province, Winnipeg and Brandon) versus rural (small communities across the province) population is approximately 72% versus 28% (Statistics Canada, 2013).

Socioeconomic status (SES) was determined on the basis of the median neighbourhood income quintiles from Statistics Canada (Statistics Canada, 2013): low income included the lowest and second lowest quintiles, high income included the three highest quintiles.

We included all Manitoba residents aged 65 years and older over an 11-year period. Separate analyses were conducted to evaluate prevalent and incident utilization of antipsychotics, BZDs, zopiclone (ZOP) and zaleplon (ZAL) in elderly persons living in the community or in PCH. SGAs available in Manitoba at the time of the study were clozapine, risperidone, olanzapine and quetiapine. Clozapine utilization in the elderly population of Manitoba has been negligible and restricted to patients with treatment-resistant-schizophrenia; therefore, it was not included in the analyses. Please refer to Appendix 1 for a complete list of BZDs and related medications. It is important to note that ZAL was discontinued in Canada by the manufacturer in September 2007 (Health Canada, 2013). Incident users were defined as individuals who were registered with the provincial health care system and had not received a prescription for the medication of interest in the year prior to the first prescription. Prevalence of use was also evaluated as defined-daily-dose (DDD) per 1,000 patients per day, which represented a measurement of intensity of use. The DDD is the average daily dose for a medication dispensed for the main indication in usual practice and is calculated according to the guidelines of the WHO Collaborating Centre for Drug Statistics Methodology (World Health Organization, 2013). The time frame of the study was between 1997/98 and 2008/09 by fiscal year (01 April to 31 March for each year). Generalized estimating equation (GEE) modeling (Vittinghoff, Glidden & Shiboski, 2012) was used to assess the influence of sociodemographic characteristics (age, sex, region of residence and SES) on the use of medications over time.

Non-optimal use definitions included incident prescription of high dose SGAs (i.e., ≥1.5 mg/day for risperidone, ≥10 mg/day olanzapine, ≥200 mg/day quetiapine) (World Health Organization, 2013) within the first year of use, use of SGAs in PCH residents with a concomitant prescription of an acetylcholinesterase inhibitor (AChEI) (donepezil, galantamine, rivastigmine) within the same year, or a concomitant prescription of an antipsychotic and a BZD, ZOP or ZAP within the same quarter. Other medications such as memantine that can be prescribed to individuals with dementia were not included in the analysis because of negligible utilization in the province and/or because of overlapping prescribing with an AChEI.

Logistic regression modeling was used to identify predictors of non-optimal use. Variables included in the model were: prescribers’ characteristics (e.g., age, sex, speciality, type of practice, hospital affiliation), patient sociodemographic characteristics and measures of health services utilization in the year prior to the first antipsychotic prescription (i.e., number of hospitalizations and ambulatory visits, number of medications, use of AChEIs, psychosis diagnosis identified by ICD9-CM 295-299, ICD–10–CM: F2, F3, F84, R410, co-morbidities as assessed by the number of major Aggregated Diagnostic Groups (ADGs), as defined in the Johns Hopkins ACG^®^ (Adjusted Clinical Group) Case-Mix System (software version 9) (Reid et al., 2013; The Manitoba Centre for Health Policy, 2013). Since time effects are important in changing prescribing patterns, optimal use evaluations were conducted in incident users between 2002/03 and 2008/09, as this was the interval that would best assess the possible effect of the warnings.

Analyses were performed using SAS^®^ statistical software, version 9.2 (SAS Institute Inc., Cary, North Carolina, USA).

## Results

### Utilization: community-dwelling population

A total of 143,491 community-dwelling adults over 65 years of age represented the population denominator in 1997/98, and 153,189 in 2008/09.

Prevalent utilization of SGAs increased from 0.6 to 13.5 users per 1,000 community-dwelling older adults while the use of FGAs declined from 12.8 to 5.9 users per 1,000. Risperidone was the most prescribed agent with an increase in prevalence from 0.6 to 6.5 per 1,000 (olanzapine use increased from 0.05 to 3.7 per 1,000 and quetiapine use increased from 0.07 to 3.9 per 1,000) during the study period. The use of BZDs was greater than the use of antipsychotics in this population but little change in prevalence was observed (108.6 to 109.1 users per 1,000); however, ZOP and ZAL use increased from 13.6 to 53.0 users per 1,000 during the same time interval. Prevalence of users receiving a combination of SGAs and BZD (or ZOP, ZAL) increased from 0.2 to 5.61 per 1,000.

Dose intensity of SGAs increased from 0.2 DDD per 1,000 in 1997/98 to a peak of 6.5 per 1,000 in 2004/05 and subsequently decreased to 4.9 by the end of the study period, 2008/09. For BZDs the change in DDD was small (from 65.2 to 68.6 per 1,000) while for ZOP and ZAL, it increased from 9.5 to 49.6 per 1,000.

Incidence rates per quarter increased significantly for SGA users and for users of combination therapy over the time period of the study. Age and gender had a significant effect with greater incident utilization in the 85 and over group compared to the 65–84 year of age group for all medications and in females compared to males for the SGAs. Higher incident use in males was observed for the combination of BZDs (or ZOP, ZAL) and SGAs (Table 1).

**Table 1:** Incident use in community-dwelling older adults (65+).

Medications	SGAs	FGAs	BZDs/ZOP, ZAL	BZDs/ZOP, ZAL + SGA
Users/1,000 Year 1998	0.21	1.88	13.14	0.06
Users/1,000 Year 2009	1.63	1.03	13.66	0.15
Change in rate per quarter	1.02^*^	0.98^*^	1.00 NS	1.01^*^
Age effect 65–84 vs. 85+	0.44^*^	0.85^*^	1.08^*^	0.60^*^
SES effect low vs. high	1.10^*^	1.14^*^	1.00 NS	1.09 NS
Region effect rural vs. urban	0.88^*^	1.51^*^	1.01 NS	1.02 NS
Sex effect male vs. female	0.91^*^	0.86^*^	0.73^*^	1.17^*^

**Notes.**

Results for change in quarterly rate, age, SES, region and sex effects are presented as relative rates (adjusted for age, SES, region, sex and time).

SGAs second generation antipsychotics FGAs first generation antipsychotics BZDs/ZOP, ZAL benzodiazepines, zopiclone, zaleplon NS not significant SES socioeconomic status

* Indicates a statistically significant effect (*p* < 0.05).

### Utilization: PCH-dwelling population

A total of 8,516 PCH-dwelling older adults represented the denominator for this population in 1997/98 and 8,818 in 2008/09.

Prevalent utilization of SGAs increased from 15.0 to 268.5 users per 1,000 PCH-dwelling older adults while the use of FGAs declined from 169.3 to 47.7 users per 1,000. Risperidone was again the most commonly prescribed agent with an increase in prevalence from 15.0 to 167.0 per 1,000 (quetiapine use increased from 0.9 to 67.6 per 1,000 and olanzapine use increased from 0.8 to 49.1 per 1,000) during the study period. While the use of BZDs declined slightly from 170.7 to 161.3 per 1,000, the use of ZOP and ZAL increased dramatically from 15.0 to 102.6 users per 1,000 during the same time. Prevalence of users receiving a combination of SGAs and BZD, ZOP or ZAL increased from 4.8 to 90.8 per 1,000.

Dose intensity of SGAs increased from 5.5 DDDs per 1,000 in 1997/98 to a peak of 85.5 per 1,000 in 2003/04 and subsequently declined to 70.7 by the end of the study period, 2008/09. For BZDs the increase in DDD was small from 82.1 to 84.9 per 1,000 between 1997/98 and 2003 with a decline to 67.0 by the end of the study period. For ZOP or ZAL, there was a significant increase from 9.5 to 49.6 per 1,000 over the entire study period.

Incidence rates per quarter increased significantly for SGAs users and for users of combination therapy over the time period of the study. Refer to Table 2 for details of the age, residence, and sex effects on incident use in this population.

**Table 2:** Incident use in personal care (nursing) home-dwelling older adults (65+).

Medications	SGAs	FGAs	BZDs/ZOP, ZAL	BZDs/ZOP, ZAL + SGA
Users/1,000 Year 1998	3.76	19.61	26.66	0.70
Users/1,000 Year 2009	21.09	8.62	22.91	4.45
Change in rate per quarter	1.01^*^	0.98^*^	1.00 NS	1.01^*^
Age effect 65–84 vs. 85+	1.21^*^	1.36^*^	1.13^*^	1.40^*^
Region effect rural vs. urban	0.78^*^	0.85^*^	0.79^*^	0.74^*^
Sex effect male vs. female	1.22^*^	1.38^*^	1.09^*^	1.48^*^

**Notes.**

Results for change in quarterly rate, age, region and sex effects are presented as relative rates (adjusted for age, region, sex and time). Individuals residing in PCH do not have values for SES.

SGAs second generation antipsychotics FGAs first generation antipsychotics BZDs/ZOP, ZAL benzodiazepines, zopiclone, zaleplon NS not significant SES socioeconomic status

* Indicates a statistically significant effect (*p* < 0.05).

### Optimal use

There were 12,878 total incident users (community and PCH-dwelling) of antipsychotic medications (FGAs and SGAs) between 2002/03 and 2007/08. Of these, 1,319 (10.2%) went on to use a high dose of a SGA within the first year of therapy.

**Figure 1:**
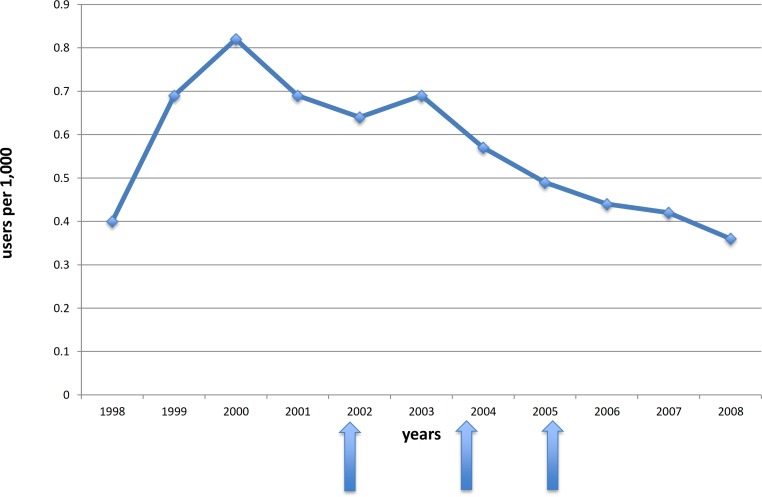
Crude incidence rates of high dose SGA use. High doses were defined as: ≥1.5 mg/day for risperidone, ≥10 mg/day olanzapine, ≥200 mg/day quetiapine. The arrows indicate the times of the warnings issued by Health Canada.

The rate of older adults who received a high dose SGA within the first year of being prescribed an SGA increased from 0.4 to 0.82 per 1,000 persons by 2000 and then declined to 0.36 per 1,000 in 2008 (Fig. 1). Among incident users of antipsychotic medications, the utilization of high dose SGAs declined from 112 to 94 per 1,000 from 2002 to 2008.

**Figure 2:**
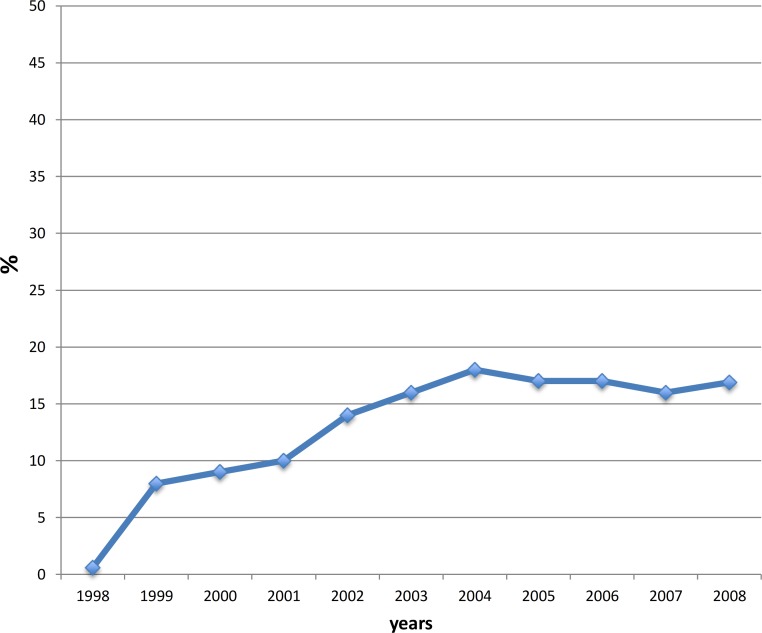
Proportion of PCH residents receiving an SGA in combination with AChEIs. PCH, personal care home (nursing home); SGA, second-generation antipsychotic; AChEI, acetylcholinesterase inhibitor.

The proportion of PCH residents who were concomitantly prescribed an SGA and an AChEI in the same year increased over time from 0.2 to 17.8% in 2004 and slightly declined to 16.9% by the end of the study (Fig. 2).

Patients were more likely to receive a high dose SGA if they were male, had received a prescription for an AChEI or had a diagnosis of psychosis within the year prior to the first prescription (Table 3). Very old patients and those treated with a greater number of different medications were less likely to receive a high dose SGA. Prescriber characteristics and use of other health care services were not significantly different.

**Table 3:** Factors predictive of high dose SGAs within the first year of use.

Variable	OR	95% CI	*P*
Age, years	0.98	0.97–0.99	<0.0001
Sex (male vs. female)	1.18	1.04–1.33	0.0095
Number of other medications	0.97	0.96–0.98	<0.0001
AChEI prescribed in the year prior to Rx	1.28	1.08–1.51	0.0041
Diagnosis for psychosis in the year prior to Rx	1.52	1.34–1.73	<0.0001

**Notes.**

ACHEI acetylcholinesterase inhibitor (donepezil, galantamine, rivastigmine) OR odds ratio Rx prescription

## Discussion

We observed a significant increase in antipsychotic utilization in this elderly Canadian population. This is consistent with other reports from Canada (Valiyeva et al., 2008; Alessi-Severini et al., 2008; The Canadian Institute for Health Information, 2009), Europe (Martinez, Jones & Rietbrock, 2013; Schulze et al., 2013) and the US (Kales et al., 2011). Greater utilization of SGAs was observed in urban elderly persons and in particular among PCH residents. In fact, a 20-fold greater utilization was observed in PCH-dwelling elderly when compared to those living in the community. By the end of the study period, more than 25% of PCH-dwelling older individuals had received an SGA. Prevalence of BZD use remained above 10% in both community- and PCH-dwelling elderly over the time period of the study, while ZOP and ZAL utilization increased significantly. We observed greater use of FGAs amongst rural community dwelling elderly, but not among rural PCH dwellers. This could possibly be explained by off-label use of FGAs, such as treatment of chemotherapy-related nausea. Another reason could be a slower adoption of the newer agents by more isolated practice sites.

It seems that prescribers in the province of Manitoba have somewhat responded to warnings about potential harm to the elderly populations from treatment with SGAs (Health Canada, 2005; Health Canada, 2002; Health Canada, 2004; The Food and Drug Administration, 2008; European Medicines Agency (EMEA), 2009): the dose intensity declined in community-dwelling and reached a plateau in PCH-dwelling residents after the warnings were issued and only a minority (approximately 10%) received potentially inappropriate high doses of antipsychotic medications. Even though a decline would have been desirable, the sign of a lower incidence of new prescriptions can be interpreted as a positive trend. As well, the proportion of new users of SGAs who were also prescribed AChEIs reached a plateau after the warnings of serious adverse events in elderly individuals with dementias were released; however, no causality can be inferred as the study was not designed to assess the effects of an intervention, but to observe changing in prescribing patterns. While some findings might be difficult to interpret, the higher use of SGAs in PCH reflects a reality of a sicker population more susceptible to psychosis and agitation secondary to dementia; moreover, the observation that males seem more likely to receive high dose SGAs can be interpreted with a higher incidence of symptoms of aggression in male dementia patients. On the other hand, the observed lower occurrence of high dose SGAs in very old patients and/or on multiple therapies appears to be consistent with appropriate prescribing to frail individuals. Clinical studies are needed to confirm these assumptions.

Limitations to measures of optimal use of prescription medications using administrative databases include the lack of clinical information regarding severity of symptoms, use of over-the-counter medications, alcohol use and parameters of quality of life. Because of the lack of clinical outcome information, this study does not allow for assessment of appropriateness in all cases; numerous clinical characteristics may indicate that medications such as high dose SGAs may in fact be appropriate therapy for a particular patient at a particular time with appropriate close monitoring and follow up.

It is important to note also that in Manitoba, up to 27% of PCHs do not have medications filled through community pharmacies (Doupe, Brownell & Kozyrskyj, 2006) and are, therefore, not captured in the DPIN system; however, the proportion of elderly patients who reside in PCHs is limited (less than 6%) and no evidence of significant differences in patients’ characteristics has been shown among all PCHs in the province (Doupe, Brownell & Kozyrskyj, 2006). As a consequence, the missing data should not introduce a selection bias nor affect the validity of the results. A strength of these analyses is, in fact, in the comprehensive nature of the administrative health claims data, which include nearly all Manitobans: our results are not affected by sampling errors or recall bias. Furthermore, all studied psychotropic medications are available as unrestricted benefits on the provincial formulary, which allowed access to medications to be unaffected by SES and reimbursement conditions. For the PCH-dwelling older adults, medications are likely administered by PCH staff, so medication adherence is assured.

In conclusion, despite encouraging trends, the use of psychotropic medications remains high in elderly individuals, especially in residents of PCH. Clinicians caring for such patients need to carefully assess risks and benefits. Current recommendations call for the use of pharmacotherapy only when psychotic symptoms and agitation are persistent, recurrent or cause clinically significant functional disruption; the lowest possible effective dose of antipsychotics should be used when necessary and a regular monitoring of efficacy, safety and tolerability should be conducted (APA Practice Guidelines, 2007; Peisah, Chan & McKay, 2011; Jeste, Blazer & Casey, 2008; Gauthier, Patterson & Chertkow, 2012).

## Appendix 1

Benzodiazepines and related medications

Alprazolam, bramazepam, chlordiazepoxide, clobazam, clonazepam, diazepam, flurazepam, lorazepam, oxazepam, temazepam, triazolam, zaleplon and zopiclone.
